# A pragmatic view on general anesthesia in mechanical thrombectomy for acute ischemic stroke

**DOI:** 10.1016/j.bjane.2025.844599

**Published:** 2025-02-18

**Authors:** Alessandro Scudellari, Federico Bilotta

**Affiliations:** a Addenbrooke's Hospital, Department of Anesthesia, Cambridge, United Kingdom; b Policlinico Umberto I, University ‘La Sapienza’, Department of Anesthesiology, Critical Care and Pain Medicine, Rome, Italy

Mechanical thrombectomy (MT) is now an established treatment for acute ischemic stroke (AIS). If the patient is cooperative and airway protection is ensured, local anesthesia or sedation can be effective strategies to facilitate MT for treating large vessel occlusion of the anterior circulation.^1^ General anesthesia (GA) was initially stigmatized due to its retrospective association with poor outcomes in large MT registries like MR CLEAN.^2^ Subsequently, randomized controlled trials (RCTs) like SIESTA and, eventually, GASS, demonstrated the non-inferiority in neurological outcomes of patients managed with GA, and there is now a consensus that GA is the way forward.^3^, ^4^, ^5^ In the evolving landscape of stroke thrombectomy, more evidence is being published on the value of conducting MT for occlusions of the more distal branches of the intracranial circulation, like the segment M2 or M3 of the middle cerebral artery and the segments A2 and A3 of the anterior cerebral artery, because they represent around a third of AIS cases. Observational data from registries show promising results in medium vessel occlusions, especially in relation to the M2 segment.^6^, ^7^, ^8^ Once completed, the trials ESCAPE-MeVO and DISTAL may be able to add robust evidence.^9^^,^^10^ Endovascular navigation to reach distal intracranial branches requires guaranteed immobility to safely reach the target clot and retrieve it, minimizing the risk of vessel injury. GA in this case may be the safest option.^11^

GA is a complex intervention that includes instrumentation of airways, administration of multiple drugs, mechanical ventilation, invasive monitoring, and above all, the significant challenge of a swift and precise interpretation of the specific pathophysiology of the treated patient. All this is in order to provide stability of all the physiologic parameters and, in particular, to support brain health during the procedure. The level of complexity increases if we consider that the targets to be achieved during GA for MT in AIS are still not established with good evidence, including blood pressure, oxygenation, and ventilation parameters.^12^ The process of delivering GA requires time and exposes the patient to additional risks. The greater the number of comorbidities a patient has, the higher the anesthetic risk. Therefore, it is essential to consider all these factors and recognize that the use of general anesthesia is not without its inherent risks. Stepping beyond the controlled environment of RCTs, the real-world clinical scenario introduces a myriad of complexities that demand a nuanced and comprehensive approach.

The foundation of medical progress often relies on evidence derived from meticulously designed RCTs, and the General Anesthesia vs Sedation for Stroke (GASS) trial has provided valuable insights confirming improved recanalization rates and functional recovery in the GA group.^4^ However, as we transition from trial settings to clinical reality, the application of these findings necessitates careful consideration of the broader healthcare landscape. Actual clinical scenarios, in stark contrast, are laden with unpredictabilities and present diverse patient profiles. Therefore, the role of the anesthesiologist becomes pivotal, extending beyond the confines of trial protocols to navigate the nuanced intricacies of individual patient care. While the GASS trial and other RCTs underscore the benefits of GA, it is equally crucial to recognize the inherent risks associated with this approach. Inadvertent hypotension, alterations in cerebral blood flow, and potential complications demand a vigilant and personalized approach to anesthetic management.

In conclusion, the recent shift in opinion regarding the safety of general anesthesia in endovascular thrombectomy is noteworthy but not definitive. The role of the anesthesiologist involves a careful evaluation of the specific patient, weighing the pros and cons, and ensuring the safest possible care.

## Conflicts of interest

The authors declare no conflicts of interest.
